# A Trauma-Informed HIV Intervention (LinkPositively) to Improve HIV Care Among Black Women Affected by Interpersonal Violence: Protocol for a Pilot Randomized Controlled Trial

**DOI:** 10.2196/46325

**Published:** 2023-07-05

**Authors:** Jamila K Stockman, Katherine M Anderson, Alexandra Fernandez DeSoto, Danielle M Campbell, Kiyomi Tsuyuki, Keith J Horvath

**Affiliations:** 1 Division of Infectious Diseases and Global Public Health Department of Medicine University of California, San Diego La Jolla, CA United States; 2 Department of Behavioral, Social, and Health Education Sciences Rollins School of Public Health Emory University Atlanta, GA United States; 3 Department of Psychology San Diego State University San Diego, CA United States

**Keywords:** HIV, peer navigation, social networking, Black women, interpersonal violence, web application, randomized controlled trial, mobile phone

## Abstract

**Background:**

Black women bear a disproportionate burden of HIV, accounting for nearly 60% of new diagnoses among US women. Black women living with HIV often experience mutually reinforcing epidemics, known as syndemics, including interpersonal violence and substance use. Syndemics are associated with decreased HIV care engagement and treatment adherence and worsening HIV outcomes. Few HIV services and resources are tailored to be culturally and gender-responsive and trauma informed for Black women living with HIV. Technology-based, psychoeducational, and peer navigation programs offer promising pathways to tailored HIV support and improved HIV care outcomes. Therefore, the web-based, trauma-informed intervention LinkPositively was developed in collaboration with Black women living with HIV to promote uptake of HIV care and ancillary support services.

**Objective:**

This study primarily determines the feasibility and acceptability of the LinkPositively intervention among Black women living with HIV affected by interpersonal violence. The secondary aim is to examine the preliminary impact of the LinkPositively intervention on retention in HIV care, antiretroviral therapy adherence, and viral suppression while evaluating the role of mechanism of change variables (eg, social support) in the associations.

**Methods:**

The LinkPositively trial is a pilot randomized controlled trial conducted in California, United States, among 80 adult Black women living with HIV who have experienced interpersonal violence. Core components of LinkPositively include one-on-one peer navigation with phone and SMS text message check-ins; 5 weekly one-on-one video sessions to build coping and care navigation skills; and a mobile app that contains a peer support social networking platform, an educational database with healthy living and self-care tips, a GPS-enabled HIV and ancillary care resource locator, and a medication self-monitoring and reminder system. Participants are randomly assigned to the intervention (n=40) or control (Ryan White standard of care; n=40) arm, with follow-up at 3 and 6 months. At each assessment, participants complete an interviewer-administered survey and submit hair samples for the assessment of HIV medication adherence. All research staff and investigators adhere to ethical principles and guidelines for conducting research activities. Data will be analyzed using generalized estimating equations.

**Results:**

Final development and testing of the LinkPositively app were completed in July 2021. As of May 2023, we have screened 97 women for eligibility. Of the 97 women screened, 27 (28%) were eligible and have been enrolled in the study. The dissemination of preliminary results will occur in 2024.

**Conclusions:**

This trial will advance HIV prevention science by harnessing technology to promote engagement in HIV care while improving social support through peers and social networking—all while being trauma informed for Black women living with HIV with experiences of interpersonal violence. If shown to be feasible and acceptable, LinkPositively has the potential to improve HIV care outcomes among Black women, a marginalized key population.

**International Registered Report Identifier (IRRID):**

DERR1-10.2196/46325

## Introduction

### Background

The United States has seen a reduction in HIV infections in recent years, yet Black women continue to bear a disproportionate burden of diagnoses [1-3]. In 2019, Black women comprised 13% of the US female population yet accounted for nearly 60% of new HIV diagnoses; by comparison, White women represented 62% of the female population but 22% of HIV diagnoses [4]. HIV incidence among Black women is 11 times that among White women (18.9 vs 1.8) [4]. Women living with HIV, in particular Black women, often experience HIV with mutually reinforcing epidemics known as syndemics. The substance use, violence, and HIV/AIDS syndemic is the overlapping and synergistic intersection of these conditions [5]; in addition, mental health coincides with the substance use, violence, and HIV/AIDS syndemic [6,7]. These mutually reinforcing conditions of violence, substance use, and adverse mental health among people living with HIV [6,7] are more prevalent among women than men [5,8-13]. Among women living with HIV, approximately 55% experience lifetime intimate partner violence (IPV) [14,15], 27% experience past-year IPV [16], and 67% experience lifetime interpersonal violence more broadly [17]. Black women generally face 35% higher lifetime IPV victimization than White women [18]. Women experiencing violence are more likely to experience adverse mental health [10,19], whereas women living with HIV have worse mental health outcomes than men living with HIV and women without HIV. Furthermore, women living with HIV experiencing IPV have worse mental health outcomes than those who do not experience IPV [19]. Black women living with HIV have 3-fold higher rates of prevalence of posttraumatic stress disorder (PTSD) and major depressive disorder compared with Black women without HIV regardless of substance use category [20]. Compared with women without HIV, women living with HIV have reported higher use of cigarettes, nonprescribed cannabis, cocaine, speed, and heroin in North American settings [21], and women living with HIV who have recently experienced violence have higher odds of using substances [22]. Together, these findings show that women living with HIV, especially Black women, are at a substantially increased risk of a variety of mental and physical health problems.

In addition to syndemic factors among women living with HIV, Black women continue to face worse HIV care outcomes than other people living with HIV, including non-Black women; these outcomes are exacerbated by syndemic experiences. Once diagnosed, Black women are significantly less likely than White women to be linked to care within 90 days of diagnosis [23], and once in care, only 56% of Black women are retained [23]. As a result, Black women are significantly less likely to be virally suppressed than White women [24,25]. Violence, substance use, and adverse mental health are associated with low or suboptimal antiretroviral therapy (ART) adherence, reduced viral suppression [10,14,15,17,22,26-29], and low retention in HIV care [30] among women living with HIV. Lifetime experiences of violence in particular are associated with an 8 times lower likelihood of being virally suppressed among women living with HIV compared with women not exposed to violence [31]. In studies that account for syndemic conditions among women living with HIV, more co-occurring conditions (IPV, substance abuse, and adverse mental health) was associated with worse ART adherence [32] and, among racial and ethnic minoritized women, reduced viral suppression [5].

Black women living with HIV lack services that are responsive to their syndemic needs, which likely contributes to suboptimal HIV outcomes. Syndemic experiences and the lack of trauma-informed care services may act as barriers to HIV care, complicating both the receipt of services logistically and the acceptability of services. People living with HIV with adverse mental health (PTSD and depression) have lower odds of retention in HIV care [30]. Furthermore, Black women living with HIV face sociostructural barriers to care (HIV stigma, medical mistrust, and unmet housing and transportation needs) at higher rates than HIV-negative and male HIV-positive individuals [33-35], affecting retention in care and ART adherence; racial and ethnic minoritized women are the most affected [34,36-42]. HIV stigma is the most noted barrier to care and ART adherence among women living with HIV [37,43-47]. Conversely, formal and informal social support networks may mitigate the effects of syndemic and sociostructural barriers on HIV care outcomes. Increased social support is associated with fewer depressive symptoms; less HIV stigma [48-50]; and improved HIV-related health symptoms [51], ART adherence [41,52-54], perceived ability to engage in HIV care [42], and HIV self-management [55].

Trauma-informed services offer a pathway to more acceptable care for syndemic-affected women living with HIV, particularly in the setting of peer navigation. Trauma-informed care is a strength-based approach that aims to meet the unique needs of trauma survivors. Trauma-informed care increases the acceptability and comfort of services while minimizing barriers to care. Although trauma-informed care has been studied and practiced in the context of addiction treatment, mental health, and health care, it has been less extensively practiced in terms of HIV prevention and treatment [56-61]. Peer navigators—medication-adherent role models with shared lived experiences with their clients—provide social support and modeling of engagement-supportive behaviors to their clients [62]. Peer navigators help build self-efficacy in patient-provider communication and decrease medical mistrust, offsetting the impact of syndemic factors affecting HIV care (eg, violence, substance use, and adverse mental health) [62]. Peer navigation for improving linkage to and retention in care among women living with HIV is feasible, acceptable, and efficacious in the United States and other countries [63-68]. Peer navigation can improve ART adherence [69,70] and viral suppression [63,71], although some studies have found no effect [72], perhaps because of the small samples or the lack of informal social support. The addition of informal social support may help reduce social isolation, build community, and share strategies for addressing barriers to ART adherence. Among women living with HIV, social support group participation with HIV-positive peers has demonstrated benefits related to mental health, relationships, and sociostructural barriers [73-76].

With increased internet access and smartphone ownership, technology-based interventions have become an increasingly feasible option for improving health [77] and demonstrate significant potential for trauma-informed interventions using peer navigation. Technology-based interventions related to HIV are primarily mobile app– or SMS text messaging–based and have been developed for women living with HIV who are outside the United States [78-81], older in the United States [82], and pregnant [83-85]; people living with HIV regardless of gender [86]; transgender women living with HIV [87,88]; and HIV prevention [89,90]. The results include decreased viral load and improved engagement in care and ART adherence [91-93]. Women report that technology-based interventions feel more confidential, lead to more efficient and perceived-safer care, and feel more empowering to women with a history of interpersonal violence than nontechnology interventions [94-98]. Overall, technology-based interventions among women living with HIV in the United States have been limited and, to our knowledge, nonexistent for Black women living with HIV or women who have experienced interpersonal violence, although most US women (91%) use the internet, including 78% of Black women [99]. Few of those that do exist have not considered the syndemic conditions and unique barriers faced by women living with HIV [100,101] despite interest in, advantages, and acceptability of technology-based interventions, particularly for increasing social support [102-105].

Given the unique needs and significant disparities faced by Black women living with HIV in the United States and the lack of services and interventions that are syndemically responsive and culturally tailored to Black women, we undertook the development of a trauma-informed HIV intervention for Black women living with HIV that uses a culturally and gender-tailored mobile app, LinkPositively, combined with one-on-one peer navigation.

### Theoretical Foundations

LinkPositively is grounded in the theory of triadic influences and syndemic theory and informed by trauma-informed care as a derivative of trauma theory. The theory of triadic influence is a multilevel social cognitive theory that understands 3 streams of influence—individual, social, and structural—that act in combination to affect health behaviors [106]. This study considers individual-level influences, such as racial or ethnic minority identity and experiences of interpersonal violence, and indirect sociostructural influences, such as medical mistrust, HIV-related stigma, and limited access to resources. In consonance, LinkPositively was created under the framework of syndemic theory, which understands HIV as co-occurring and mutually exacerbating with other comorbidities in disadvantaged populations [107]. These synergistic conditions are influenced by the social, economic, environmental, and political settings in which a population exists [107]. Within the population of interest (Black women living with HIV who have experienced interpersonal violence), these other syndemic conditions or experiences include violence, mental health disorders, and substance use, which, in combination, negatively and synergistically affect HIV care outcomes. Finally, trauma-informed care is a service delivery model that aims to be responsive to the impacts of trauma by emphasizing physical, psychological, and emotional safety; empowerment; and healing for survivors of trauma and their care providers [108]. According to trauma theory, unprocessed trauma is stored as physiological reactions to circumstances that recall the initial trauma [109]; health care interactions in particular may elicit trauma responses [110]. Evidence from mental health, substance use, and social service settings finds that adopting trauma-informed care practices improves patient outcomes and enhances patient satisfaction and engagement with clinic staff [111]. Simultaneously, there have been calls to integrate trauma-informed care practices within HIV care settings [112,113].

### Objectives

The primary aim of this study is to determine the feasibility and acceptability of the LinkPositively intervention and mobile app among Black women living with HIV affected by interpersonal violence. The secondary aim is to examine the preliminary impact of the LinkPositively intervention on retention in HIV care, ART adherence, and viral suppression while evaluating the role of mechanism of change variables (ie, social support, activation of social support networks, self-efficacy, and the use of ancillary support services) in the associations. Collectively, both aims serve as preparation for a large-scale R01 randomized controlled trial.

## Methods

### Study Design

**Figure 1:**
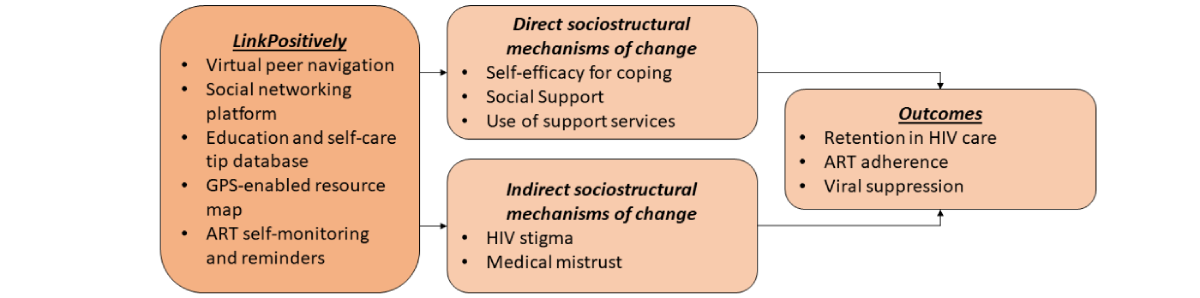
LinkPositively conceptual model. ART: antiretroviral therapy.

The LinkPositively trial is a pilot randomized controlled trial of 80 Black women living with HIV who have experienced lifetime interpersonal violence, with 6 months of follow-up after enrollment. Women are randomized to the LinkPositively intervention (intervention arm; n=40) or Ryan White standard of care (control arm; n=40) groups. Survey and biological assessments occur at baseline and 3 and 6 months after randomization to evaluate improvement in primary and secondary outcomes. Feasibility and acceptability measures are assessed using a combination of self-reported surveys, intervention paradata, and study process measures (eg, retention rates). The primary outcomes are retention in HIV care, ART adherence, and viral suppression. Secondary outcomes are hypothesized direct and indirect mechanisms of change, including self-efficacy for coping, social support, the use of ancillary support services, HIV stigma, and medical mistrust (Figure 1).

### Study Population

The inclusion criteria for LinkPositively are self-reported (1) self-identification as a woman; (2) Black or African American racial and ethnic background; (3) age of ≥18 years; (4) HIV-positive status; (5) current residence in the San Diego, Los Angeles, or Alameda counties in California, United States; (6) linked to HIV care but having fallen out of care; (7) lifetime experience of physical (ie, hitting, slapping, kicking, assault with a weapon, or threats thereof), sexual (ie, attempted, forced, or threatened sexual activities, including unwanted touching), or psychological (ie, yelling or insulting, controlling behavior, and fear of violence) abuse by a current or former partner or nonpartner (eg, relative, friend, or stranger); (8) ownership of a smartphone with internet-browsing capabilities; and (9) ability to speak English. Being out of care is defined as not having had a blood test while taking HIV medication for at least 3 months or within the past year, not having had a medical appointment with an HIV care provider, not having had an ART prescription filled, or not having had blood tests to monitor HIV (CD4 counts and HIV viral load). The exclusion criteria are (1) unwillingness to participate in the study and (2) cognitive impairment that would limit participation in study procedures. The study staff assess cognitive impairment through direct observation. Specifically, if during the consent process a participant appears to be experiencing paranoia or auditory hallucinations, has sporadic outbursts, or is not capable of comprehending the interview or materials, the participant is excluded from the trial.

### Study Procedures

#### Recruitment and Screening

The recruitment methods for LinkPositively include 3 main approaches. First, we partnered with the San Diego Center for AIDS Research Health Equity Sociobehavioral Science Core to distribute flyers to community-based agencies. We also used the recruitment and retention subcommittee of the San Diego Center for AIDS Research Health Equity Sociobehavioral Science Core Community Advisory Board to promote and advertise the study. We partnered with local health care clinics and HIV service organizations within the community to passively recruit through flyer distribution. Los Angeles–based recruitment used the Los Angeles County Service Planning Area provider meetings; Commission on HIV caucus and work group meetings; and local community advisory board meetings, support groups, and education forums. Alameda County–based recruitment used the Collaborative Community Planning Council of the Oakland Transitional Grant Area and local HIV service agencies to disseminate study flyers. Second, we used social media platforms to maximize outreach and recruitment in the 3 California counties. Social media platforms included Facebook, Instagram, and Twitter. Paid advertisements, promotions, and web banner advertisements will be used to target populations based on demographic characteristics, including gender, age, race, ethnicity, and location of residence. Finally, we distributed advertisements, newspaper advertisements, and flyers at bus stops, libraries, transitional housing facilities, social service agencies, and businesses located in San Diego, Los Angeles, and Alameda County communities with at least 10% Black residents. Recruitment and screening for the study are ongoing to facilitate the development of a study cohort consisting of approximately 80 eligible women.

#### Enrollment and Randomization

Women interested in participating in LinkPositively provide consent to be screened and complete a screening survey that includes questions regarding age, gender, self-reported HIV status, HIV care status, and violence exposure history. The screening survey is self-administered or staff-administered depending on the preference of the potential participant and will be housed in REDCap (Research Electronic Data Capture; Vanderbilt University). Eligible participants are invited to enroll in LinkPositively and scheduled for an in-person or on the web (via Zoom [Zoom Video Communications]) baseline enrollment visit. At the baseline study visit, the participant provides documented informed consent and completes a 45-minute interviewer-administered, computer-assisted personal interview hosted on REDCap on topics including sociodemographic characteristics, HIV care, treatment history, substance use, mental health status, interpersonal violence, care interactions, medical mistrust, coping self-efficacy, social support, stigma, COVID-19, and technology use (Table 1). Participants also provide a hair sample in person or via mail to assess for tenofovir and emtricitabine concentrations as an objective measure of ART adherence. The hair sample collection kits include 1 piece of aluminum foil, 2 white adhesive labels, 2 alcohol swabs, 1 small freshness packet, and scissors. Hair sample collection involves the following steps: (1) cleaning the blades of a pair of scissors with an alcohol pad, (2) lifting up the top layer of hair from the occipital region of the scalp to isolate a small thatch of hair (approximately 100 fibers of hair) from underneath the top layer, (3) cutting the small hair sample as close to the scalp as possible, (4) keeping fingers on the part of the hair that was furthest away from the scalp and placing the hair sample down on an unfolded piece of tin foil, (5) placing a thin label over the end of the hair sample that was furthest away from the scalp, (6) refolding the foil over to completely enclose the hair and placing a study identification number label on the folded piece of foil, and (7) placing the folded piece of foil inside the plastic bag and sealing the bag. All hair samples are securely shipped to the University of California, San Francisco Hair Analytical Laboratory, where liquid chromatography or tandem mass spectrometry will be used to assess antiretroviral concentrations in hair samples over the past 3 months, requiring 3 cm of hair. Participants are compensated with US $25 upon the completion of the survey and US $25 upon the provision of the hair sample.

**Figure 2:**
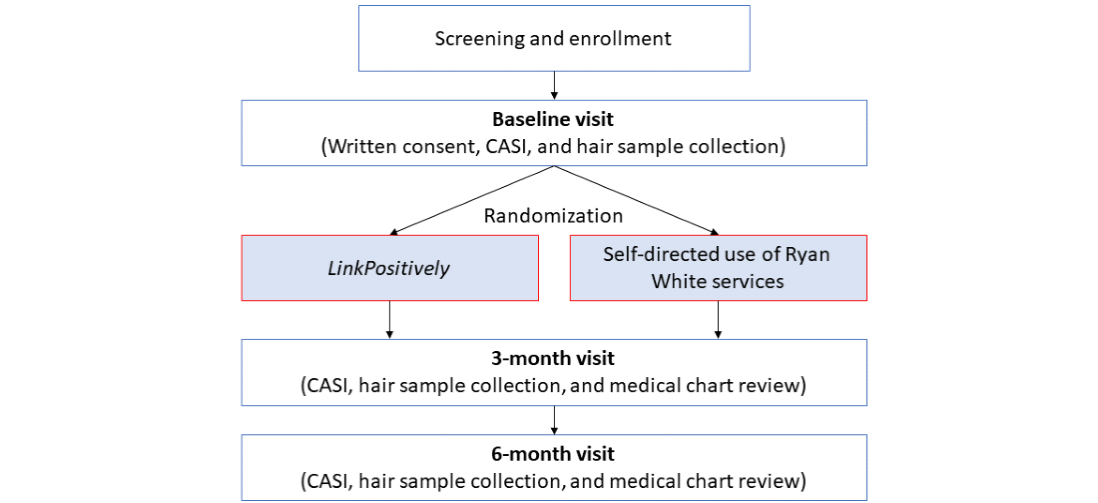
Study flow. CASI: Computer Assisted Self-Interview.

Following the completion of the baseline survey, participants are randomized at a 1:1 ratio into the LinkPositively intervention group or the control group, stratified by perpetrator of violent experience (intimate partner vs nonintimate partner). Randomization is conducted using a randomly permuted block randomization design by a study staff member with no participant contact. Participants are informed of their group assignment, and participants assigned to the LinkPositively intervention arm are shown how to download the LinkPositively app and given a demonstration of LinkPositively features. Follow-up assessments occur at the 3- and 6-month follow-up visits, requiring a survey and hair sample collection (Figure 2).

**Table 1:** Exposure and outcome measures.

Measure category		Measures
**Evaluation outcomes**		
	Feasibility	Number of participants recruited, number of participants enrolled, number of participants retained, and number of intervention group sessions completed
	Acceptability	CSQ-8^a^ and System Usability Scale [114]
**Individual-level factors (survey)**		
	Sociodemographic characteristics	Age, education, employment, living situation, income, relationship status, household composition, medical insurance status, and food insecurity (6 items) [115]
	HIV care and treatment history	Year first diagnosed with HIV, year they first started medical care for HIV, usual place for HIV treatment (yes or no), frequency of seeing an HIV care provider, period of intermittent care (yes or no), taking antiretroviral medication (yes or no), HIV medication adherence [116] (3 items), and barriers to HIV treatment since diagnosis [117] (14 items)
	Interpersonal violence	Intimate partner violence (12 items), revised Conflict Tactics Scale (ever and since last visit) [118]; non–intimate partner violence (physical, sexual, or psychological abuse by a relative, friend, or stranger [ever and since last visit]); ACEs^b^ (10 items); mental health coercion (3 items); substance use coercion (3 items); and DA-5^c^ (5 items)
**Co-occurring syndemic conditions (survey)**		
	Mental health	Depression (10 items), PHQ-9^d^ [119]; PTSD^e^ (9 items), NSESS^f^-PTSD [120]; anxiety (7 items), GAD-7^g^ [121]; stressful life experiences (20 items), PCL-5^h^ [122], and traumatic life events (17 items), LEC-5^i^ [123]; resilience (10 items), PLHIV^j^ Resilience Scale [124]; and suicidality (4 items), SBQ-R^k^ [125]
	Substance use	Alcohol use (10 items), AUDIT-10^l^ [126], and drug use, NIDA^m^-modified ASSIST^n^ 2.0 [127]
**Direct sociostructural mechanisms of change measures (survey)**		
	Coping self-efficacy	Self-Efficacy Scale (CSE^o^, 26 items) [128]
	Social support	MMOS-SS^p^ (20 items) [129]
	Ancillary support use	Use and met need of ancillary support services (eg, transportation, housing, mental health care, domestic violence services, and substance use treatment)
**Indirect sociostructural mechanisms of change measures (survey)**		
	HIV stigma	Chronic Illness Anticipated Stigma Scale (30 items) [130]
	Medical mistrust	GBMMS^q^ (12 items) [131]
	COVID-19–specific PTSD	5 items, adapted PC-PTSD-5^r^ [132]
**Outcome measures**		
	Retention in HIV care	Having ≥2 laboratory tests (CD4^s^ or VL^t^) dated at least 90 days apart [133]
	ART^u^ adherence	Self-reported 3-item measure, dichotomized (>90% adherent vs <90% adherent) [116]
	ART adherence (objective measure)	Tenofovir and emtricitabine concentrations in hair sample [134]
	Viral suppression	Undetectable or <200 copies of viral DNA per mL vs detectable or ≥200 copies of viral DNA per mL [135]

^a^CSQ-8: Client Satisfaction Questionnaire–8 item.

^b^ACE: adverse childhood experience.

^c^DA-5: Danger Assessment Short Form.

^d^PHQ-9: Patient Health Questionnaire–9 item.

^e^PTSD: posttraumatic stress disorder.

^f^NSESS: National Stressful Events Survey PTSD Short Scale.

^g^GAD-7: Generalized Anxiety Disorder–7.

^h^PCL-5: Posttraumatic Stress Disorder Checklist- 5 Item.

^i^LEC-5: Lifetime Events Checklist DSM 5.

^j^PLHIV: people living with HIV.

^k^SBQ-R: Suicidal Behaviors Questionnaire-Revised.

^l^AUDIT-10: Alcohol Use Disorders Identification Test-10 Items.

^m^NIDA: National Institute on Drug Abuse.

^n^ASSIST: Alcohol, Smoking and Substance Involvement Screening Test.

^o^CSE: Coping Self-Efficacy Scale.

^p^MMOS-SS: Modified Outcomes Study Social Support Survey.

^q^GBMMS: Group-Based Medical Mistrust Scale.

^r^PC-PTSD-5: Primary Care PTSD Screen for DSM-5 (PC-PTSD-5).

^s^CD4: cluster of differentiation 4.

^t^VL: Viral Load.

^u^ART: antiretroviral therapy.

### Intervention Group

LinkPositively is a trauma-informed peer navigation intervention using a mobile-optimized web application that aims to improve retention in HIV care and ART adherence and promote reaching or maintaining viral suppression among Black women living with HIV who have experienced interpersonal violence. The development of the LinkPositively app with Black women living with HIV has been previously described [136]. In brief, the app comprises a social networking platform to facilitate connections among Black women living with HIV and information sharing, with the option to do so anonymously; an educational and self-care tip database; a self-monitoring and reminder system to promote ART adherence and other health behaviors; and a GPS-enabled resource locator for local and national HIV and ancillary care services (Table 2). Women assigned to the LinkPositively intervention group have access to all components of the LinkPositively app and one-on-one peer navigation. At the baseline visit, the women are introduced to the LinkPositively app. The study staff conduct onboarding, which involves training participants on how to download the app onto their smartphone, explaining the 5 components, and using each component, with an emphasis on contacting their assigned peer navigator. Within the first week of participants being assigned to this group, peer navigators schedule and complete a one-on-one video intake session with the participant. During this one-on-one intake session, the peer navigator conducts a thorough participant needs assessment to connect the participant to HIV medical care via local health clinics and identify other areas of need, services of need, and assisted referrals. These areas include but are not limited to domestic violence services, mental health care, substance abuse treatment, and housing and legal support. Peer navigators provide trauma-informed, intensive emotional and informational support, including guidance on accessing information (eg, agency name, address, website, and contact information) about referred services. Participants attend five 60-minute web-based one-on-one peer navigation sessions over a 5-week period, covering a variety of topics: building coping skills for syndemic conditions, activating social support networks, and addressing medical mistrust (Table 3). In addition, participants text or call their peer navigators during and after the 5-week period if they have questions or seek to debrief after sessions or appointments. In the back end of the web application, peer navigators enter case notes on peer navigation sessions and check-ins, set notifications and appointment reminders, and send their assigned participants direct messages that show up only on that participant’s profile. Peer navigators can also view the women’s responses in real time and data on medication and appointment outcomes to inform their upcoming session or check-in. In addition to videoconference or phone sessions and check-ins, participants receive automated daily medication reminder SMS text messages, scheduled appointment alerts, and access to daily postings in the education and self-care tip component of the app.

**Table 2:** LinkPositively app components, features, and mechanisms of change.

App element	Features	Hypothesized mechanism of change influenced
Profile page	Option to customize avatar from a variety of preloaded options or upload photo^a^ Option to share information in the “about me” section Option to customize profile page background using preloaded optionsa	N/A^b^
Social networking	Ability to post text, images, or videos to a “timeline”^a^ Ability to react to content using emoticons Ability to comment on others’ postsa	Social support HIV stigma
Education and self-care tips	Rotating tip featured daily on the timeline Searchable database of tips categorized using tags Integrated external resources	Coping self-efficacy Ancillary support use HIV stigma Medical mistrust
ART^c^ self-monitoring and reminders	Pre-established monitoring of ART adherence and mood Ability to create customized reminders Option to receive reminders at the time of their choosing Option to receive reminders via in-app notifications or SMS text message Ability to view responses to tracked activity in a past-month calendar depiction	Coping self-efficacy
Resource locator	Searchable database of services related to HIV and non-HIV medical and social support services Up-to-date information on location, hours, and contact information Information on insurance acceptance when available	Coping self-efficacy Ancillary support use Medical mistrust

^a^Indicates features that may require *unlocking* through continued app use.

^b^N/A: not applicable.

^c^ART: antiretroviral therapy.

**Table 3:** LinkPositively intervention content delivered via one-on-one peer navigation sessions.

Session number	Topic	Objectives
1	Emotional awareness	Understanding how trauma and other co-occurring conditions (eg, substance use) affect emotions Learn ways in which social environments influence how we experience emotions (social influences on our emotional experience exercise) Introduce and practice labeling feeling states Introduce and practice focused breathing
2	Emotional regulation and distress tolerance	Introduce the benefits of emotion regulation (common unhealthy coping skills exercise and handout) Learn the 3 ways in which we experience emotion (the 3 areas of emotion exercise or handout) Explain why we tolerate unpleasant emotions and how to know whether to tolerate the unpleasant emotions or not Learn how to assess the pros and cons of distress tolerance (pros and cons list) Introduce different methods for healthy coping (with distress) with 3 ways in which we experience emotions (positive self-statements, pleasurable activities, and formal time-out)
3	Relationship patterns and activating social support networks	Introduce interpersonal or relationship patterns (exercise—what are interpersonal patterns? The self-fulfilling prophecy) Identify feelings and interpersonal or relationship patterns (how to identify your interpersonal patterns exercise and common interpersonal patterns exercise) Learn what assertive communication and behavior are (exercise in defining assertive, nonassertive, and aggressive communication and behavior) Understand how assertive behavior works in healthy relationships (healthy boundaries) Learn how to make requests and ask for help clearly and in an assertive manner Understand when and how to say “no” Introduce basic personal rights (exercise and handout)
4	Interpersonal violence and the impact on existing sociostructural barriers	Identify ways in which power and control dynamics, disclosure of violence, and interpersonal violence influence HIV stigma and medical mistrust Explain the 3 types of power balances in relationships (exercise and handout) Identify personal relationship patterns Explore flexibility in relationships Understand the role compassion and respect play in how we view ourselves and others
5	Medical mistrust and review	Understand the relationship between discrimination and medical mistrust Review conspiracy theories and HIV treatment Learn skills for effective patient-provider communication Review material covered in all 5 sessions

### Control Group

Women assigned to the control arm are connected with and receive self-directed (not peer navigation–supported) treatment as usual at agencies and institutions in the San Diego, Los Angeles, or Alameda counties following the Ryan White HIV/AIDS Program standard of care (ie, referrals to physical, dental, and mental health care services; medical case management; and ancillary services [eg, alcohol/substance use recovery and family support]). Women who are not already receiving services and do not have other means of receiving services or are not under medical case management elsewhere are referred to care. Alternatively, women may request case management services via the Ryan White HIV/AIDS Program elsewhere in the counties.

Using the Ryan White standard of care, goals are set to create an individual care plan related to medical care, housing, financial, and other resources as needed. Referrals are made to appropriate services (eg, primary care, housing, benefits counseling, transportation, food, legal services, and support services) based on an intake interview. Women who require a higher level of care (eg, significant mental health concern, at risk, or falling out of care) and do not have other means of receiving services or are not under medical case management elsewhere may request case management services via the Ryan White HIV/AIDS Program.

Participants requiring medical case management have a case file that provides appropriate participant follow-up and tracking information. Case managers have in-person contact with all participants at least every 90 days to assess the further need for services. Either the participant or the case manager initiates a given contact. The case manager has in-person or phone contact with participants at least every 30 days to discuss changes and progress toward meeting the goals of the individual care plan and an in-person meeting with each participant at least once every 90 days. Participants are reassessed at least once every 120 days. It is important to note that the medical case management approach is self-guided versus intense peer navigation assistance, meaning that participants learn the responsibility to move toward autonomy in their HIV care.

### Follow-Up

Participants enrolled in LinkPositively have 2 follow-up visits at 3 and 6 months after their baseline visit. Although follow-up visits occur virtually through Zoom, participants still follow the same activities from their baseline visit. Activities include a 45-minute computer-assisted follow-up survey and a self-collected hair sample. Participants are compensated with US $25 upon the completion of the survey and US $25 upon the provision of the hair sample.

### Safety Protocols

Given that LinkPositively’s focus is on women who have experienced interpersonal violence, we took additional measures to ensure the safety of participants in the study. This included the creation of a safety protocol, wherein all participants are screened for current IPV and risk of homicide by a current partner using the Danger Assessment [137] and are provided with violence-related resources, which are integrated both within the LinkPositively app and on the LinkPositively website. Furthermore, participants are asked about safety information when the study staff contacts them, such as the use of a different name or only contacting the participant through certain means or at certain times, and hair sample collection is conducted at a safe location of the participant’s choosing. For participants who indicate current experiences of violence, the study team offers a warm handoff to violence services and follows up with the participant to ensure their safety. Finally, safety elements are integrated into the LinkPositively app itself, including an automatic log-out feature when the app is swiped away from and an ambiguous app icon and home screen that disclose no information about the purpose of the app and do not reference HIV. Study staff also monitor the social networking feed on the app to identify posts that suggest potential harm to the self or others. These posts are flagged, and a study staff member follows up with the participant, activating the safety protocol and reporting this as an adverse event to the University of California, San Diego (UCSD) Human Research Protection Program (HRPP). To date, no adverse events have been reported to the UCSD HRPP.

### Ethics Approval, Informed Consent, and Data Privacy

All study procedures were reviewed and approved by the UCSD HRPP (UCSD HRPP project 191398), and a reliance agreement was executed for San Diego State University and Charles R. Drew University of Medicine and Science to rely upon the UCSD HRPP determination. Before engaging with research participants, all study staff members completed training in the ethical conduct of human participant research, data management and compliance, and the Health Insurance Portability and Accountability Act (HIPAA). All women enrolled in LinkPositively provide written informed consent and are asked to sign a HIPAA authorization form. This study was issued a certificate of confidentiality by the National Institutes of Health.

### Data Management and Quality Assurance

In compliance with National Institute of Mental Health policies, LinkPositively operates under a data and safety monitoring plan, which details methods for secure data collection, management, and monitoring. Under the data and safety monitoring plan, all digital data collected are stored on a password-protected encrypted server, whereas physical copies of data are retained in a locked filing cabinet in a locked office within a restricted-access research building. Identifying information is stored separately from the study data, with study records associated via a participant identification number. Data for linkage between participant identification number and identifying information are stored in a HIPAA-compliant secure web application designed for participant management and tracking (Ripple Science). Study staff members are only provided with data access commensurate with their study role and specifically the lowest level of access needed. Access to study data is not granted until after the completion of full ethical training. Deidentified quantitative data are downloaded on a weekly basis by the study data manager. Hair samples are associated with participant identification numbers and stored in a locked cabinet within a locked office until they are batch shipped for analysis at the University of California, San Francisco. Following completion of the analyses, the results are transmitted deidentified via secure email to UCSD.

The study staff actively monitor adverse events and serious adverse events, which are reported immediately to the principal investigator and within 24 hours to the UCSD HRPP. Finally, study activities are monitored by a Data and Safety Monitoring Board (DSMB). The DSMB convenes twice annually, at which time the study team provides updates on study activities and the DSMB provides recommendations for participant safety and data security. The DSMB comprises (1) a professor of social work and licensed social worker with substantial experience in the development and implementation of interventions for gender-based violence and HIV, (2) a professor of disease prevention and health promotion and board-certified registered nurse with substantial research experience in health information use among people living with and at risk of HIV, and (3) a biostatistician with substantial experience in randomized controlled trials in public health research.

### Analysis

#### Aim 1

The first aim is to determine the feasibility and acceptability of LinkPositively. We will assess the feasibility of the intervention by computing the percentage of participants retained at each follow-up visit and chi-square tests or Fisher exact tests to compare the intervention and control groups. Retention cutoff percentages will be used to determine the level of feasibility as follows: (1) ≥90%=strong feasibility, (2) 80% to 89%=acceptable feasibility, (3) 70% to 79%=modest feasibility with a need for improvement, and (4) <70%=unacceptable. We will assess the acceptability of the intervention by computing the percentage of participants endorsing a particular level of satisfaction on a Likert scale from questions on the Client Satisfaction Questionnaire–8 [138] and System Usability Scale [139]. We will obtain these percentages at the 3- and 6-month follow-up assessments. Responses from women in the intervention and control groups will be compared using 2-tailed *t* tests (for continuous acceptability measures) and chi-square tests (for dichotomous acceptability measures). We will also compute similar percentages based on responses from the peer navigators at the 6-month follow-up time point.

#### Aim 2

The second aim is to examine the preliminary impact of LinkPositively. Self-reported dichotomous outcomes of retention in HIV care, ART adherence, and viral suppression will be evaluated. Outcomes will be past–3-month measures assessed at each time point (3- and 6-month follow-ups). The primary independent variable will be intervention group. Chi-square tests will be used to test for significant differences in self-reported outcomes by intervention group. We will use generalized estimating equations to generate 3 independent repeated-measure logistic regressions to model retention in care, self-reported ART adherence, and viral suppression. We will also examine an objective biological outcome measure of ART adherence based on tenofovir and emtricitabine hair concentrations. We will use univariable random-intercept linear regression models to model tenofovir and emtricitabine hair concentrations, with the final model based on a generalized estimating equation repeated-measure linear regression model with a time variable (3 and 6 months). Aligned with the theory of triadic influence and syndemic theory, we will assess the impact of individual- (eg, sociodemographics, HIV treatment history, and social support), syndemic- (eg, PTSD and substance use), social- (eg, medical mistrust and HIV-related stigma), and structural-level (eg, unstable housing and insurance coverage for HIV services) factors on dependent variables. During model building, we will consider variables associated with outcome variables (univariate: *P*<.10; bivariate: *P*<.10). For the final models, adjusting for type-I error, the significance level will be set at *P*<.05.

Secondary analyses will be conducted among the 40 women randomized to the intervention group. We will estimate the association between the individual features of the LinkPositively app and retention in care, ART adherence, and viral suppression. Individual features of the LinkPositively app will include (1) the frequency of participant initiation of peer navigation through texting or calling the peer navigator, (2) number of messages sent and viewed to or by peers using the social networking platform, (3) frequency of viewing educational and self-care tips, (4) number of times the GPS-enabled resource locator was accessed, and (5) number of points earned through gamification features. Individual features of the LinkPositively app will be independent variables, and retention in care and ART adherence measures will be dependent variables. The regression models will follow those outlined for the primary analyses.

## Results

LinkPositively was approved by the institutional review board in October 2019 and funded in December 2019. The LinkPositively app was designed and developed between February 2020 and January 2021. Beta testing and refinement of the app were completed in July 2021. Recruitment began in February 2022, with the goal of enrolling 3 participants per month. As of April 2023, we have screened 97 women for eligibility and enrolled 27 (28%) eligible participants. We anticipate the dissemination of preliminary results in early 2024.

## Discussion

### Anticipated Main Findings

LinkPositively is the first intervention that combines a tailored mobile app and peer navigation to address HIV care outcomes among Black women living with HIV who have a lifetime history of interpersonal violence. The anticipated main finding of this intervention study is that LinkPositively will be feasible and acceptable for Black women living with HIV. It is also anticipated that Black women living with HIV randomized to the LinkPositively arm will demonstrate improvements in retention in HIV care, ART adherence, and viral suppression compared with Black women living with HIV randomized to the control arm.

The use of a virtual intervention overcomes a number of structural- (eg, stigma) and individual-level (eg, access to transportation) barriers to intervention engagement. This is particularly important as our study population—Black women living with HIV—experience structural barriers (eg, medical mistrust and discrimination) to engagement with HIV health services [140,141]. In addition, social support provided by both the peer navigators and other study participants target interpersonal-level facilitators, reinforcing resilience and connectedness, which have been shown to be associated with improved HIV care outcomes. In addition, the peer navigators help disseminate and change behavioral norms that are conducive to achieving viral suppression. The integration of peer navigation, an evidence-based practice, may support further improvements in Black women’s overall health and well-being outcomes beyond the HIV care continuum.

### Future Research

If shown to be feasible and acceptable, LinkPositively has the potential to serve as an alternative for this marginalized, key population. We will test it further in a larger efficacy trial among a broader sample of Black women living with HIV. Although LinkPositively may not be accessible to all Black women living with HIV who experience interpersonal violence as some may not have access to a smartphone, it represents a critical avenue for intervention to improve HIV care among a large and growing group of women and has the potential for scale-up.

## Appendix

Multimedia Appendix 1 Grant peer review report.

## Abbreviations

ART: antiretroviral therapy
DSMB: Data and Safety Monitoring Board
HIPAA: Health Insurance Portability and Accountability Act
HRPP: Human Research Protection Program
IPV: intimate partner violence
PTSD: posttraumatic stress disorder
REDCap: Research Electronic Data Capture
UCSD: University of California, San Diego

## Data Availability

Data sharing is not applicable to this paper as no data sets were generated or analyzed during this study.
