# Supplementary material for: A Trauma-Informed HIV Intervention (LinkPositively) to Improve HIV Care Among Black Women Affected by Interpersonal Violence: Protocol for a Pilot Randomized Controlled Trial
Source: JMIR Res Protoc. 2023 Jul 5;12:e46325. doi: 10.2196/46325 (PMC10357377; doi:10.2196/46325)
Supplement: Multimedia Appendix 1 [file resprot_v12i1e46325_app1.pdf]

SUMMARY STATEMENT

(Privileged Communication)

PROGRAM CONTACT:
Theresa Senn
301-761-7852
teri.senn@nih.gov

Release Date: 08/18/2019
Revised Date:

Application Number: 1 R34 MH122014-01

Principal Investigators (Listed Alphabetically):

HORVATH, KEITH JOSEPH
STOCKMAN, JAMILA KINSHASA (Contact)

Applicant Organization: UNIVERSITY OF CALIFORNIA, SAN DIEGO

Review Group: ZRG1 AARR-N (53)
Center for Scientific Review Special Emphasis Panel
RFA-MH20-200: Addressing the Role of Violence on HIV Care
AIDS

Meeting Date: 07/19/2019
Council: OCT 2019
Requested Start: 12/01/2019
RFA/PA: MH20-202
PCC: 9A-ASGP

Project Title: LinkPositively: A Technology-Delivered Peer Navigation and Social Networking Intervention to Improve HIV Care Across the Continuum for Black Women Affected by Interpersonal Violence
SRG Action: Impact Score:26
Next Steps: Visit https://grants.nih.gov/grants/next_steps.htm
Human Subjects: 30-Human subjects involved - Certified, no SRG concerns
Animal Subjects: 10-No live vertebrate animals involved for competing appl.
Gender: 1A-Both genders, scientifically acceptable
Minority: 1A-Minorities and non-minorities, scientifically acceptable
Age: 1A-Children, Adults, Older Adults, scientifically acceptable

Project Year	Direct Costs Requested	Estimated Total Cost
1	150,000	237,354
2	150,000	237,354
3	150,000	237,354
TOTAL	450,000	712,061

1R34MH122014-01 Stockman, Jamila

RESUME AND SUMMARY OF DISCUSSION: In this application, the investigators propose a randomized controlled trial to assess the preliminary impact of an intervention, LinkPositively, on improving HIV treatment outcomes among violence-affected Black women living with HIV in San Diego, California. The mobile health intervention will consist of virtual peer navigation via phone, text, and video; social networking; an educational and self-care tips database; GPS for location of support services; and antiretroviral therapy self-monitoring and reminders. Black women compared to other racial/ethnic groups are less likely to engage and be sustained in HIV care. Addressing factors that contribute to this risk is of clear public health importance. Some panel members however, voiced concern about the unsustainability of efforts given the required resources. The investigative team is collectively strong covering the necessary scientific expertise and experience to successfully conduct the proposed research. The multimodal intervention strategies consisting of peer navigation and technology is novel in this population context. The scientific rigor of the research approach is modest. Strengths of the approach include the incorporation of a community advisory board and theory to inform efforts, the solid leveraging of preliminary data, the strong qualitative methodology to support study phase I, the objective measurement of primary outcomes, and the robust data analysis plan. These strengths are countered by the inattention to differential effects due to intervention delivery format, the insufficient cultural tailoring of strategies, and concerns about randomization parameters. Despite the weaknesses, the significance of the proposed research, investigative team, and innovation sustained high enthusiasm for improving HIV treatment outcomes among violence-affected Black women living with HIV.

DESCRIPTION (provided by applicant): In the US, Black women living with HIV/AIDS (WLHA) are less likely to be engaged in care, adherent to antiretroviral therapy (ART), and virally suppressed compared to White WLHA. Concurrently, Black women are also disproportionately affected by interpersonal violence – physical, sexual, and/or psychological abuse by a current or former intimate partner or non-intimate partner – which may co-occur with poor mental health and/or substance use disorders, further contributing to ART non-adherence, lower CD4 counts, and reduced viral suppression. Peer Navigation, while highlighted as a successful model of care in improving HIV care outcomes, requires resources that HIV service agencies often lack. A scalable and sustainable solution is the use of mobile health (mHealth) smartphone applications ("apps"). Although there has been an increase in mHealth interventions developed for HIV prevention and care among at-risk and HIV-positive MSM and youth, we are unaware of any to improve retention in care, ART adherence, and viral suppression among Black WLHA, nor any mHealth interventions that are responsive to Black women's experiences with interpersonal violence. To address this gap, we will develop and pilot test a culturally tailored, trauma-informed smartphone app, called LinkPositively, for Black WLHA affected by interpersonal violence. Core components of LinkPositively include: a) Virtual Peer Navigation that includes phone and text check-ins and 4 weekly one-on- one video sessions to build skills to cope with barriers and navigate care; b) Social Networking platform to receive peer support; c) Educational and Self-care database with healthy living and self-care tips; d) GPS- enabled Resource Locator for HIV care and ancillary support service agencies; and e) ART self-monitoring and reminder system. Guided by the Theory of Triadic Influences and Syndemic Theory, the study will be conducted in 2 phases with corresponding aims. In Aim 1, 4 focus groups with Black WLHA with experiences of interpersonal violence, one focus group with peer navigators, and 4-6 key informant interviews with providers will be conducted to determine which app features, content, and functions are most likely to support downloading, initiating use, and sustaining engagement over time. Aim 1 will culminate in usability testing by Black WLHA affected by interpersonal violence (n=5), to finalize intervention components and procedures. In Aim 2, we will pilot test LinkPositively to assess feasibility and acceptability and determine preliminary effects of the intervention on HIV care outcomes (i.e., retention in care, ART adherence, viral suppression) and mechanism of change variables (i.e., social support, self-efficacy). Participants will be randomly assigned to either the intervention (n=40) or control (Ryan White standard

of care, n=40) arm, with follow-up at 3- and 6- months. This study will benefit the advancement of HIV prevention science by harnessing technology to promote engagement in HIV care, while improving social support through peers and social networking – all under the auspices of being trauma-informed for Black WLHA with experiences of interpersonal violence.

PUBLIC HEALTH RELEVANCE: Black women living with HIV/AIDS (WLHA) who are also impacted by interpersonal violence and co-occurring mental health and/or substance use disorders, suffer from poor outcomes along the HIV care continuum. Although peer navigation improves HIV care outcomes, this requires resources that HIV medical and service organizations often lack, calling for the need for more sustainable and scalable approaches. We propose to develop and pilot test LinkPositively, a culturally-tailored, trauma-informed smartphone app for Black WLHA with lifetime experiences of interpersonal violence, with the goal of improving retention in care, antiretroviral therapy adherence, and viral suppression.

CRITIQUE 1

Significance: 2
Investigator(s): 2
Innovation: 1
Approach: 3
Environment: 1

Overall Impact: The proposed application is to develop and pilot test LinkPositively, a culturally-tailored, trauma-informed smartphone app for Black women living with HIV (WLHA) with lifetime experiences of interpersonal violence, with the goal of improving retention in care, anti-retroviral therapy adherence, and viral suppression. Core components of the program include virtual peer navigation, social networking, healthy living and self-care tips, GPS-enabled resource locator for HIV care, and ART self-monitoring and reminder system. The program is guided by the Theory of Triadic Influences and Syndemics and the study will be conducted in two phases. The first will be a developmental phase whereby qualitative approaches will be used to help inform intervention development and usability testing will be conducted to finalize intervention components and procedures. The second phase will consist of a randomized controlled trial to assess the feasibility, acceptability and impact of the intervention. Primary study outcomes are retention in HIV care, ART adherence, and viral suppression. Secondary outcomes described as socio-cultural mechanism of change variables are social support and activation of social support networks, self-efficacy, and utilization of ancillary support services. Strengths of the study include the intended audience as a critical population of interest that experiences high rates of violence, the collective expertise of the MPI team of Stockman and Horvath, the innovative methodologies and novel theoretical concept to be tested. The proposed study has the potential to lay the foundation for a scalable mHealth intervention to address the HIV treatment needs of Black women impacted by interpersonal violence (IPV)and HIV.

1. Significance:

Strengths

- The investigators present clear evidence that Black women are disproportionately affected by HIV and IPV.
- The investigators note potential correlations between violence and other psychosocial factors that commonly occur with violence.

- The scientific rationale and need for a clinical trial to execute the proposed study aims is well supported by extant literature.
- The proposed study has the potential to lay the foundation for a scalable mHealth intervention to address the HIV treatment needs of Black women impacted by IPV and HIV.

Weaknesses

- There is limited focus on the different types of violence exposures that will be addressed in the study.

2. Investigator(s):

Strengths

- The investigative team is comprised of experienced, interdisciplinary collaborators in the areas of HIV prevention and treatment, mHealth intervention development, epidemiology, psychology (specifically PTSD, trauma, and loss), and statistics.
- The prior work of the MPIs is complementary and will contribute to the success of the proposed project.

Weaknesses

- There is no evidence of extensive prior collaboration between the MPIs.

3. Innovation:

Strengths

- The investigators identify several aspects of the proposed work that contribute to the innovative nature of the project: (1) pilot testing an mHealth intervention for Black WLHA who have experienced interpersonal violence; (2) incorporating multiple components in the intervention, namely virtual peer navigation, social networking, education and self-care tips, resource locator, and ART self-monitoring and reminders; (3) addressing structural barriers to healthcare seeking via GPS resource locator; (4) testing a new conceptual model to address the co-occurrence of interpersonal violence, co-morbidities, and socio-cultural barriers to the HIV care continuum. This reviewer agrees.

Weaknesses

- None noted.

4. Approach:

Strengths

- The overall strategy is clearly articulated, sufficiently thorough, and well-reasoned to achieve the study aims.
- Qualitative assessments of phase 1 including input from the CFAR Disparities Core CAB is an asset to the proposed work.

Weaknesses

- For the randomized controlled trial phase of the study, the control condition is a self-directed usual care treatment. It is unclear how the investigators will be sure that any differences noted

between conditions are due to the content of the interventions rather than the delivery method (i.e. face-to-face usual care versus mHealth intervention).

- The investigators identify the critical need for culturally-relevant, gender-specific, mhealth interventions tailored for Black WLHA; however, the intervention content or components do not exploit this need. This is a significant shortcoming of the proposed approach.

5. Environment:

Strengths

- Structural support (i.e., facilities and resources) for the proposed work is sufficient. Additionally, investigative team members have successfully recruited and retained participants using similar study approaches.

Weaknesses

- None noted.

Study Timeline:

Strengths

- Timeline is adequate for the proposed work.

Weaknesses

- None noted.

Protections for Human Subjects:

Acceptable Risks and/or Adequate Protections

- Protections for human subjects are adequate.

Data and Safety Monitoring Plan (Applicable for Clinical Trials Only):

Acceptable

- Data and safety monitoring plan is acceptable.

Inclusion Plans:

- Sex/Gender: Distribution justified scientifically
- Race/Ethnicity: Distribution justified scientifically
- For NIH-Defined Phase III trials, Plans for valid design and analysis:
- Inclusion/Exclusion Based on Age: Distribution justified scientifically
- Distribution of sex/gender, race/ethnicity, and age are scientifically justified.

Vertebrate Animals:

Not Applicable (No Vertebrate Animals)

Biohazards:

Not Applicable (No Biohazards)

Resource Sharing Plans:

Acceptable

- Resource sharing plan is acceptable as detailed.

Authentication of Key Biological and/or Chemical Resources:

Not Applicable (No Relevant Resources)

Budget and Period of Support:

Recommend as Requested

CRITIQUE 2

Significance: 3
Investigator(s): 1
Innovation: 3
Approach: 4
Environment: 2

Overall Impact: This is a well written proposal by a strong investigative team that aims to address an important public health issue: retention in care and clinical outcomes for Black and African women who are HIV positive. There are several innovative aspects of the proposal and it is motivated by compelling previous studies. Questions in the approach, particularly in terms of how the intervention content would be tailored to the target population; and potential issues of significance dampened enthusiasm slightly.

1. Significance:

Strengths

- Black and African American women are disproportionately impacted by HIV in the United States. Improving retention to care, medication adherence, and sustained viral load suppression for this subpopulation could have public health impact.

Weaknesses

- That participants need to be compensated for their data use during the study raises concerns about intervention feasibility in the ‘real world’.
- While hiring the 2 PNs full time increases the likelihood of success for the proposed R34, what are the potential implications for sustainability longer term?

2. Investigator(s):

Strengths

- This is a very strong research team. MPI Horvath has extensive experience developing and testing technology-based interventions related to HIV behaviors. MPI Stockman has extensive research experience working with HIV positive Black and African American women, including Peer Navigation programs.

Weaknesses

- More could be said about how the MPIs have collaborated previously.

3. Innovation:

Strengths

- Given the lack of tailored programming for HIV positive Black and African American women, the target population is innovative.
- The combined use of an automated website and digital peer support is innovative as well.
- The use of hair screening to measure adherence is somewhat novel.

Weaknesses

- None noted.

4. Approach:

Strengths

- The intervention development process is stepwise and well described.
- Although not required for an R34, the previous studies motivating the current proposal are compelling and demonstrate reason for optimism for the current proposal.
- The use of a mobile-optimized website, as opposed to an app, increases the number of people who can access it.
- The plans to gamify the experience to encourage ongoing engagement with the program are well described and interesting.
- The training of PNs is well described.
- The aim of the focus groups and planned discussion topics are well articulated.
- Objective measures are proposed for the clinical outcomes. The plans to operationalize feasibility and acceptability are also well described.
- Program use data will be examined to explore whether different program components were used more, and how usage relates to program impact.

Weaknesses

- The description of the 4 structured PN sessions is useful but it's unclear how the topics map to the intervention underpinnings articulated at the top of page 75. More broadly, more could be said about how the intervention will be specific to experiences of Black and African American women.
- It might be helpful to include people from the target population during the design and development of the intervention. While the CAB will be consulted, they might have different feedback than the women. And while the women are included in the beta test, it usually is too late to make bigger changes that could have easily been integrated during the design phase.
- Measurement of the main outcomes could be better justified. It's unclear why both self-report and clinic / biological data are being used to measure the same constructs. It's also unclear what data will be abstracted from the medical charts – on page 80, it says viral load; on page 82, it says retention in care
    - Also, the measure for retention in care may need to be reconsidered. It is described as at least 2 lab tests dated at least 90 days apart, but it also says that outcomes will be the post 3 months (i.e., 90 days; pg. 82).

- Apps and mobile websites generally have low engagement over time. it would be useful if the researchers spoke a bit more about their previous experiences promoting engagement, not just retention in the study.
- Also, while it is a core aspect of the gamification experience, it would be helpful if the researchers could speak to any potential implications of providing more – and potentially more relevant – content (e.g., videos) to those who are more engaged. Does this in some way give those who are more likely to engage in care greater access, while excluding less engaged women from features they might find useful?
- Given the stratification of women based upon the perpetrator type in the FGs and beta test, I was surprised that this was not included as a stratification variable in the randomization. It also would be helpful to provide a brief justification about why perpetration type is the important factor that creates the most heterogeneity of experience among women who have been victims of IPV
- It would be helpful if the researchers talked briefly about the potential implications of including women who have a lifetime history as opposed to recent history of IPV
- Female gender is an eligibility criterion. Please clarify whether this means that both cisgender female and trans women will be eligible.
- As part of the secondary analyses, it might be useful to examine the number of PN meetings the participant attended (Range: 0-4) in addition to the number of self-initiated outreaches they made to the PN
- While it’s clear that the CAB will be a useful source of recruitment, an important piece of the flow seems to be missing. It’s unclear if people will in the beta test and pilot RCT will be referred to the study team, if flyers will be made available in waiting areas, if providers will be asked to discuss the study with their patients, etc. In short: how do attendees of these CBOs learn about the study?

5. Environment:

Strengths

- The environment can well support the proposed research.
- The CAB provides a proven resource to support recruitment goals.

Weaknesses

- None noted.

Study Timeline:

Strengths

- The timeline is feasible.

Weaknesses

- None noted.

Protections for Human Subjects:

Acceptable Risks and/or Adequate Protections

Data and Safety Monitoring Plan (Applicable for Clinical Trials Only):

Acceptable

Inclusion Plans:

- Sex/Gender: Distribution justified scientifically
- Race/Ethnicity: Distribution justified scientifically
- For NIH-Defined Phase III trials, Plans for valid design and analysis: Not applicable
- Inclusion/Exclusion of Children under 18: Excluding ages <18; justified scientifically

Vertebrate Animals:

Not Applicable (No Vertebrate Animals)

Biohazards:

Not Applicable (No Biohazards)

Resource Sharing Plans:

Acceptable

Authentication of Key Biological and/or Chemical Resources:

Not Applicable (No Relevant Resources)

Budget and Period of Support:

Recommend as Requested

CRITIQUE 3

Significance: 2
Investigator(s): 1
Innovation: 2
Approach: 4
Environment: 1

Overall Impact*:* This R34 application seeks to develop and pilot test a culturally tailored, trauma-informed smartphone app, called LinkPositively, for Black WLHA affected by interpersonal violence. Core components of LinkPositively include: a) Virtual Peer Navigation that includes phone and text check-ins and 4 weekly one-on-one video sessions to build skills to cope with barriers and navigate care; b) Social Networking platform to receive peer support; c) Educational and Self-care database with healthy living and self-care tips; d) GPS enabled Resource Locator for HIV care and ancillary support service agencies; and e) ART self-monitoring and reminder system. Investigators will conduct focus groups with Black WLHA with experiences of interpersonal violence, peer navigators, and conduct key informant interviews with providers will be conducted to determine which app features, content, and functions are most likely to support downloading, initiating use, and sustaining engagement over time. Investigators will then conduct usability testing by Black WLHA affected by interpersonal violence (n=5), to finalize intervention components and procedures. Investigators will pilot test LinkPositively to assess feasibility and acceptability and determine preliminary effects of the intervention on retention in care, ART adherence, viral suppression. 80 participants will be randomly assigned to either the intervention

(n=40) or control (Ryan White standard of care, n=40) arm, with follow-up at 3- and 6- months. This proposal is highly responsive to the RFA and has many strengths including focus on understudied and underserved population of Black women living with HIV and experiencing violence. However, while exciting and innovative it is not entirely clear from the application that an app-based intervention is currently perceived as needed, helpful, acceptable, or something these women anticipate using. More preliminary data on the perceived likelihood of use would have greatly bolstered this well-written application.

1. Significance:

Strengths

- Focus on HIV CoC outcomes among Black women experiencing interpersonal violence is well justified and warranted.
- Review of both social-structural barriers and buffering effect of social support is a strength.
- Provide evidence and justification for success of prior peer navigation programs in improving HIV CoC outcomes.
- Application largely responsive to RFA.

Weaknesses

- While substance use and mental health disparities are discussed it is not clear how focal these targets will be in the development of the app-based intervention over and above the focus on violence. A presentation of prior app-based interventions that address substance use and mental health among populations affected by HIV would have bolstered this section.
- Not clear how app will address stigma and medical mistrust among Black women experiencing violence. A review of how prior app-based interventions have addressed these barriers and the strengths/weakness and rigor of the prior projects would have greatly

2. Investigator(s):

Strengths

- PIs have content expertise and previous experience leading similar NIH-funded projects.
- Preliminary evidence presented is comprehensive and bolsters confidence that the investigators can carry out the proposed project in a culturally competent and appropriate way.

Weaknesses

- None noted.

3. Innovation:

Strengths

- First app-based intervention tailored for Black women living with HIV.
- Integration of peer navigation on a mHealth platform is novel for Black women living with HIV.
- GPS technology for easy location of culturally competent, trauma-focused, HIV, mental health, substance use, and HIV providers as well as other resources for Black women experiencing violence is a strength.

Weaknesses

- Not clear from application how innovative app-based peer navigation is.
- App functions and features while, innovative, seem quite ambitious.

4. Approach:

Strengths

- Use of existing CFAR CAB is a strength.
- Focus on addressing violence and other related syndemic conditions is a strength.
- Proposed design appropriate for R34 mechanism and highly responsive to RFA.
- Use of Tridic and Syndemic as guiding theories are strengths in this application.
- Adherence measures (hair samples) are appropriate, acceptable, and less invasive than traditional blood draws.
- Proposed survey measures are appropriate and comprehensive.
- Analysis plan is solid and appropriate for meeting study aims.

Weaknesses

- 6-month follow up period is less than ideal time-frame for assessing long-term results but OK for R34.
- App functions and features seem quite ambitious and will not be possible to determine which components are most responsible for any observed changes in CoC outcomes.
- A major goal of app is linkage to ancillary support services. Will be difficult to delineate any observed changes in CoC outcomes due to app features and functions over and above the utilization of these ancillary support services.
- It is not clear that Black women living with HIV and experiencing interpersonal violence see need for an app-based intervention. Any data on perceived need and acceptability, and perceived likelihood of use would greatly bolster this application.
- Not clear how app will address stigma and medical distrust specifically.
- Not clear how much of a focus app will have on substance use and the mental health needs of these women.
- Extent to which virtual peer navigators will have capacity and training to deliver trauma-informed care is questionable.
- Extent to which these women would engage in social networking on this app rather than using existing apps like Facebook or Instagram is questionable.

5. Environment:

Strengths

- UCSD and SDSU provide excellent resources and environment conducive to conducting the proposed project.

Weaknesses

- None noted.

Study Timeline:

Strengths

- Timeline is comprehensive and appropriate for carrying out the proposed project.

Weaknesses

- None noted.

Protections for Human Subjects:

Acceptable Risks and/or Adequate Protections

- Proper plans to reduce harm and maximize benefits to participants.

Data and Safety Monitoring Plan (Applicable for Clinical Trials Only):

Acceptable

- Appropriate plans to safeguard data and participants in place.

Inclusion Plans:

- Sex/Gender: Distribution justified scientifically
- Race/Ethnicity: Distribution justified scientifically
- For NIH-Defined Phase III trials, Plans for valid design and analysis: Not applicable
- Inclusion/Exclusion Based on Age: Distribution justified scientifically
- Inclusion criteria justified and appropriate given the focus.

Vertebrate Animals:

Not Applicable (No Vertebrate Animals)

Biohazards:

Not Applicable (No Biohazards)

Resource Sharing Plans:

Acceptable

Authentication of Key Biological and/or Chemical Resources:

Not Applicable (No Relevant Resources)

Budget and Period of Support:

Recommend as Requested

THE FOLLOWING SECTIONS WERE PREPARED BY THE SCIENTIFIC REVIEW OFFICER TO SUMMARIZE THE OUTCOME OF DISCUSSIONS OF THE REVIEW COMMITTEE, OR REVIEWERS' WRITTEN CRITIQUES, ON THE FOLLOWING ISSUES:

PROTECTION OF HUMAN SUBJECTS: ACCEPTABLE

INCLUSION OF WOMEN PLAN: ACCEPTABLE

INCLUSION OF MINORITIES PLAN: ACCEPTABLE

INCLUSION ACROSS THE LIFESPAN PLAN: ACCEPTABLE

COMMITTEE BUDGET RECOMMENDATIONS: The budget was recommended as requested.

Footnotes for 1 R34 MH122014-01; PI Name: Stockman, Jamila Kinshasa

NIH has modified its policy regarding the receipt of resubmissions (amended applications). See Guide Notice NOT-OD-14-074 at http://grants.nih.gov/grants/guide/notice-files/NOT-OD-14-074.html. The impact/priority score is calculated after discussion of an application by averaging the overall scores (1-9) given by all voting reviewers on the committee and multiplying by 10. The criterion scores are submitted prior to the meeting by the individual reviewers assigned to an application, and are not discussed specifically at the review meeting or calculated into the overall impact score. Some applications also receive a percentile ranking. For details on the review process, see http://grants.nih.gov/grants/peer_review_process.htm#scoring.

MEETING ROSTER

Center for Scientific Review Special Emphasis Panel
CENTER FOR SCIENTIFIC REVIEW
RFA-MH20-200: Addressing the Role of Violence on HIV Care

ZRG1 AARR-N (53)
07/19/2019

Notice of NIH Policy to All Applicants: Meeting rosters are provided for information purposes only. Applicant investigators and institutional officials must not communicate directly with study section members about an application before or after the review. Failure to observe this policy will create a serious breach of integrity in the peer review process, and may lead to actions outlined in NOT-OD-14-073 at https://grants.nih.gov/grants/guide/notice-files/NOT-OD-14-073.html and NOT-OD-15-106 at https://grants.nih.gov/grants/guide/notice-files/NOT-OD-15-106.html, including removal of the application from immediate review.

CHAIRPERSON(S)

YBARRA, MICHELE L, PHD
CEO AND RESEARCH DIRECTOR
CENTER FOR INNOVATIVE PUBLIC HEALTH RESEARCH
SAN CLEMENTE, CA 92672

MEMBERS

BALAN, IVAN C, PHD
ASSOCIATE PROFESSOR
DEPARTMENT OF PSYCHIATRY
COLUMBIA UNIVERSITY
NEW YORK, NY 10032

BASS, JUDITH KAREN, PHD
ASSOCIATE PROFESSOR
DEPARTMENT OF MENTAL HEALTH
BLOOMBERG SCHOOL OF PUBLIC HEALTH
JOHNS HOPKINS UNIVERSITY
BALTIMORE, MD 21205

BIELLO, KATIE BROOKS, PHD
ASSOCIATE PROFESSOR
DEPARTMENT OF BEHAVIORAL AND SOCIAL SCIENCES
DEPARTMENT OF EPIDEMIOLOGY
SCHOOL OF PUBLIC HEALTH
BROWN UNIVERSITY
PROVIDENCE, RI 02912

BOURIS, ALIDA M, PHD
ASSOCIATE PROFESSOR
CENTER FOR HEALTH ADMINISTRATION STUDIES
SCHOOL OF SOCIAL SERVICE ADMINISTRATION
UNIVERSITY OF CHICAGO
CHICAGO, IL 60637

CHRISTOPOULOS, KATERINA A, AB, MD, MPH
ASSOCIATE PROFESSOR
HIV/AIDS DIVISION
SAN FRANCISCO GENERAL HOSPITAL
UNIVERSITY OF CALIFORNIA, SAN FRANCISCO
SAN FRANCISCO, CA 94110

DARBES, LYNAE A, PHD
ASSOCIATE PROFESSOR
DEPARTMENT OF HEALTH BEHAVIOR
AND BIOLOGICAL SCIENCES
SCHOOL OF NURSING
UNIVERSITY OF MICHIGAN
ANN ARBOR, MI 48109

EATON, LISA A, PHD
ASSOCIATE PROFESSOR
DEPARTMENT OF HUMAN DEVELOPMENT
AND FAMILY STUDIES
COLLEGE OF LIBERAL ARTS AND SCIENCES
UNIVERSITY OF CONNECTICUT
STORRS, CT 06029

HOUSTON, ERIC, PHD
ASSISTANT PROFESSOR
DEPARTMENT OF PSYCHIATRY AND HUMAN BEHAVIOR
CHARLES R DREW UNIVERSITY OF MEDICINE
AND SCIENCE
LOS ANGELES, CA 90059

INGRAM, LUCY ANNANG, PHD
ASSOCIATE PROFESSOR
DEPARTMENT OF HEALTH PROMOTION, EDUCATION,
AND BEHAVIOR
ARNOLD SCHOOL OF PUBLIC HEALTH
UNIVERSITY OF SOUTH CAROLINA
COLUMBIA, SC 29208

JOHNSON, DAWN M, PHD
ASSOCIATE PROFESSOR
DEPARTMENT OF PSYCHOLOGY
UNIVERSITY OF AKRON
AKRON, OH 44325

LELUTIU-WEINBERGER, CORINA, PHD
ASSISTANT PROFESSOR
RUTGERS BIOMEDICAL AND HEALTH SCIENCES
SCHOOL OF NURSING
RUTGERS UNIVERSITY
NEWARK, NJ 07101

LUSENO, WINFRED K, PHD
RESEARCH SCIENTIST
PACIFIC INSTITUTE FOR RESEARCH AND EVALUATION
CHAPEL HILL, NC 27514

MUNOZ-LABOY, MIGUEL A, DPH
ASSOCIATE PROFESSOR
SCHOOL OF SOCIAL WORK
COLLEGE OF PUBLIC HEALTH
TEMPLE UNIVERSITY
PHILADELPHIA, PA 19122

PENCE, BRIAN W, PHD
ASSOCIATE PROFESSOR
DEPARTMENT OF EPIDEMOLOGY
GILLINGS SCHOOL OF GLOBAL PUBLIC HEALTH
UNIVERSITY OF NORTH CAROLINA
CHAPEL HILL, NC 27516

RAJ, ANITA, PHD
PROFESSOR
DIVISION OF GLOBAL PUBLIC HEALTH
DEPARTMENT OF MEDICINE
CENTER ON GENDER EQUITY AND HEALTH
UNIVERSITY OF CALIFORNIA, SAN DIEGO
LA JOLLA, CA 92093

SMITH FAWZI, MARY CATHERINE, SCD
INSTRUCTOR AND EPIDEMIOLOGIST
DEPARTMENT OF GLOBAL HEALTH AND SOCIAL MEDICINE
HARVARD MEDICAL SCHOOL
BOSTON, MA 02115

STORHOLM, ERIK DAVID, PHD
BEHAVIORAL SCIENTIST
RAND CORPORATION
SANTA MONICA, CA 90401

WELLES, SETH L, PHD, SCD, MD, DSC
PROFESSOR AND CHAIR
DEPARTMENT OF EPIDEMIOLOGY AND BIOSTATISTICS
SCHOOL OF PUBLIC HEALTH
DREXEL UNIVERSITY
PHILADELPHIA, PA 19102

ZIMMERMAN, RICK S, PHD
PROFESSOR
COLLEGE OF NURSING
LOUISANA STATE UNIVERSITY
ST. LOUIS, MO 63121

SCIENTIFIC REVIEW OFFICER

BYNUM, SHALANDA A, PHD
SCIENTIFIC REVIEW OFFICER
CENTER FOR SCIENTIFIC REVIEW
NATIONAL INSTITUTES OF HEALTH
BETHESDA, MD 20892

EXTRAMURAL SUPPORT ASSISTANT

STROTHERS, DIARA
EXTRAMURAL SUPPORT ASSISTANT
CENTER FOR SCIENTIFIC REVIEW
NATIONAL INSTITUTES OF HEALTH
BETHESDA, MD 20892

Consultants are required to absent themselves from the room during the review of any application if their presence would constitute or appear to constitute a conflict of interest.
